# MALAT1 knockdown alleviates the pyroptosis of microglias in diabetic cerebral ischemia via regulating STAT1 mediated NLRP3 transcription

**DOI:** 10.1186/s10020-023-00637-2

**Published:** 2023-04-03

**Authors:** Nan Zhao, Wei Hua, Qi Liu, Yueying Wang, Zhiyi Liu, Sinan Jin, Benshuai Wang, Yuxin Pang, Jiping Qi, Yuejia Song

**Affiliations:** 1 Department of Pathology, First Clinical Hospital, Harbin Medical University, No. 23 Post Street, Nangang District, Harbin, 150001 Heilongjiang China; 2 Department of Endocrinology, First Clinical Hospital, Harbin Medical University, No. 23 Post Street, Nangang District, Harbin, 150001 Heilongjiang China

**Keywords:** Diabetic cerebral ischemia, MALAT1, Pyroptosis, STAT1, Cerebral ischemic reperfusion

## Abstract

**Background:**

Dysregulated long non-coding RNAs participate in the development of diabetic cerebral ischemia. This study aimed to investigate the underlying mechanism of lncRNA MALAT1 in diabetic cerebral ischemia.

**Method:**

Middle cerebral artery occlusion (MCAO) was performed to establish diabetic cerebral I/R in vivo. TTC and neurological deficits assessment were performed to assess cerebral ischemic injury. LDH was conducted to detect cytotoxicity. RT-qPCR and western blotting assays were applied to determine mRNA and protein expression. Flow cytometry was performed to detect the pyroptosis of BV2 cells. Immunofluorescence and FISH were conducted for subcellular localization of MALAT1 and STAT1. ELISA was performed to determine cytokine release. Dual luciferase reporter, RIP, and ChIP assays were used to validate the interaction between STAT1 and MALAT1/NLRP3. Diabetes aggravated cerebral injury in vivo and in vitro. Diabetic cerebral ischemia induced inflammatory response and inflammation-induced cell pyroptosis.

**Result:**

MALAT1 was overexpressed in diabetic cerebral ischemia models in vivo and in vitro. However, knockdown of MALAT1 suppressed inflammatory response and the pyroptosis of BV2 cells. Moreover, MALAT1 interacted with STAT1 to transcriptionally activate NLRP3. Knockdown of STAT1 significantly reversed the effects of MALAT1. Furthermore, STAT1 promotes the MALAT1 transcription. MALAT1 interacts with STAT1 to promote the pyroptosis of microglias induced by diabetic cerebral ischemia through activating NLRP3 transcription.

**Conclusion:**

Thus, knockdown of MALAT1 may be a potential promising therapy target for diabetic cerebral ischemia.

**Supplementary Information:**

The online version contains supplementary material available at 10.1186/s10020-023-00637-2.

## Introduction

Cerebral ischemia is featured by high mortality and disability rates. Over 30% of ischemic patients are diagnosed with diabetic disease (Park and Koh 2018). Diabetes is reported to be an independent risk factor for stroke; meanwhile, the morbidity of cerebral ischemia patients with diabetic disease is 2 to 4 times to the patients without (Shupletsova et al. 2017; Li et al. 2004; Ding et al. 2005). Inflammation is closely related to the occurrence and development of cerebral ischemia, mainly involving brain edema, the destruction of the blood–brain barrier, the infiltration of inflammatory cells, and the expression of adhesion molecules (Xie et al. 2020). Additionally, the inflammation have been shown to correlate with atherosclerotic process, cardiovascular events, and acute ischemic cerebrovascular syndrome (Della Corte et al. 2016; Tuttolomondo et al. 2008; Pinto et al. 2006). However, the specific mechanism is still unclear. The current therapies for cerebral ischemia are chiefly based on increasing perfusion, but it might develop into ischemia/reperfusion (I/R) injury (Luan et al. 2013). Therefore, to further explore therapeutic approaches to ameliorate cerebral ischemic injury is a pivotal issue.

Pyroptosis, also known as inflammatory cell death, is a programmed cell death first identified by Brennan and Cooksen in 2000 (Brennan and Cookson 2000). It is triggered by a series of pattern recognition receptors (PRRs): activated NLRP3 inflammasomes, amino-terminal gasdermin D (GSDMD-N) with pore-forming activity. Next, the activated GSDMD-N binds to the plasma membrane and forms large oligomeric pores, releasing cell contents and pro-inflammatory factors (IL-1β, IL-18). Inflammatory response is an essential pathogenesis of diabetes. Furthermore, pyroptosis expands the inflammatory effects and participates in the progression of numerous diabetic complications, including periodontitis (Zhou et al. 2020), nephropathy (Zhan et al. 2020), retinopathy (Yu et al. 2021) and cardiomyopathy (Xu et al. 2020). Tu et al. (2019) revealed that the pyroptosis of microglias in diabetic patients is remarkably increased compared with the non-diabetic ischemic stroke. Therefore, the pyroptosis of microglias makes limited contributions to the establishment of the diversity, stability and plasticity in brain nervous system.

Long noncoding RNAs (lncRNAs) are involved in various physiology and pathology progressions, including pyroptosis. With the deepening research of lncRNAs, lncRNA Metastasis Associated Lung Adenocarcinoma Transcript 1 (MALAT1) has been revealed to aggravate ischemic stroke via the MDM2/p53 (Zhang et al. 2018, 2020; Wang et al. 2019). Besides, MALAT1 performed regulatory functions on pyroptosis in diabetic complications. For instance, knockdown of MALAT1 exhibited suppressive effects on macrophage pyroptosis in rats with diabetic atherosclerosis (Han et al. 2018). In diabetic nephropathy (DN), deletion of MALAT1 restrained pyroptosis in high-glucose-treated HK-2 cells (Li et al. 2017a; Liu et al. 2020), suggesting the regulatory functions of MALAT1 in pyroptosis signaling pathway. However, its potential roles and underlying in diabetic cerebral ischemia-related pyroptosis remain unknown.

In the present study, we aim to elucidate that MALAT1 participated in the pathogenesis of the diabetic cerebral I/R injury via MALAT1/STAT1-mediated pyroptosis of microglias.

## Material and methods

### Animal model

Sixty db/db rats and forty BALB/c mice (weigh: 20 ± 2 g; sex: male; age: 8–10 weeks) were purchased from the Nanjing Medical University. The mice were raised in a dry and ventilated pathogen-free barrier facility with 60% relative humidity, 12 h/d lighting time at 25 °C with free access to food and water. After acclimatization for 2 days, mice were randomly divided into five groups: normal group, cerebral I/R group (n = 20; both using C57BL/6 mice), diabetes group, diabetic cerebral I/R group (n = 20; both using db/db mice) and diabetic cerebral I/R + MALAT1 short hairpin RNA (sh-MALAT1) group (n = 20; using db/db mice). The mice in cerebral I/R group and diabetic cerebral I/R group were anesthetized using 1% pentobarbital sodium (50 mg/kg), followed by performed to establish I/R model using middle cerebral artery occlusion (MCAO) method (Wang and Zhou 2018). Briefly, mice were anesthetized by intraperitoneal injection of 10% chloral hydrate (3.5 ml/kg). The the mice were fixed in the supine position and incised a 1.5 cm cut in the middle cervical region to expose and separate the right common carotid artery (CCA), internal carotid artery (ICA) and external carotid artery (ECA). Subsequently, the proximal part of CCA and the distal end of ECA were ligated. The opposite site of CCA was clamped. A bullet headed monofilament nylon suture was inserted into the ECA until a slight resistance was obtained. After 2 h, the suture was removed to restore blood flow. The mice of the other two groups were modeled following the same procedure as above. At 36 or 72 h after reperfusion, neurological deficit score of each mouse was evaluated according to the criteria of Longa scoring standard (Longa et al. 1989): 0—no neurological deficit symptoms; 1—unable to extend the left fore limb while lifting the tail; 2—circling toward the left side; 3—difficult to walk, fall toward the left side; 4—impaired in walk and unconsciousness. Whereafter, the mice were sacrificed by intraperitoneal injection of pentobarbital sodium (150 mg/kg) and the brain tissues were resected for the subsequent experiment. The brain tissues were promptly stored in liquid nitrogen. All animal experiments were supervised by the Ethics Committee of First Clinical Hospital, Harbin Medical University.

### Intracerebroventricular injection

Ad-sh-MALAT1 was synthesized by Shanghai GenePharma Co., Ltd. As previously described (Su et al. 2017), the MALAT1 knockdown group mice were placed on a stereotactic frame, and received intracerebroventricular injection at bregma with 6 μl Ad-sh-MALAT1 (4.0 × 10^8^ IU) 20 min before I/R modeling.

### The encephalaedema volume

After removing the lower brain stem and epencephalon, the brain tissue was sliced into 2 mm-thickness slides. The slides were incubated with 2% TTC solution in the dark room at 37 °C for 20 min. Next, 4% paraformaldehyde solution was used to fix the slides for 24 h. The images were photographed and analyzed using Image J (ver. 1.37C). The volume of encephaledema was calculated according to the formula: V = ∑(A1 + A2)t/2. V: volume of encephaledema (mm^3^); A1 and A2: front and back encephaledema area of the slides (mm^2^); t: thickness of the slides (mm).

### Cell hypoxia/reoxygenation (H/R) model

Cells from the mice brain tissues were isolated as previously described (Zhou et al. 2016). BV2 cell line was purchased form ATCC, USA and recovered in our lab. Cells were divided into control group, high-glucose group, H/R group and high-glucose + H/R group. To establish the H/R cell model, the cells were cultured in glucose-free DMEM and incubated at 37 °C in a multi-gas incubator with 94% N_2_, 5% CO_2_ and 1% O_2_. Following hypoxia, Cells were incubated for another 4 h in standard DMEM at 37 °C with 5% CO_2_ for reoxygenation.

### Cell transfection

Cell transfection was carried out 2 h after the cell modeling. MALAT1 small interfering RNA (si-MALAT1), si-STAT1, plasmid harboring MALAT1 (pcDNA3.1/MALAT1) and their negative controls were designed and synthesized by NovaBio Co., Inc. and transfected into the cells using Lipofectamine® 2000 reagent (Invitrogen). After transfection, the cells were maintained in high-glucose (50 mM) DMEM or normal DMEM for 72 h.

### PI staining assay

Cell death was determined using PI One-Step Staining Assay Kit (G2680; Solarbio) according to the manufacturer's manual. Briefly, cells were resuspended in PBS at the density of 1 × 10^6^/ml. 95 μl of the cell suspension and 5 μl of PI were incubated for 5 min in shade. Subsequently, PI-positive cells were photographed under a fluorescent microscope (DM4B; Leica Inc.).

### Lactate dehydrogenase (LDH) analysis

The amount of LDH was measured using a LDH Assay Kit (C0017; Beyotime) followed the manufacturer’s manual. The medium was collected and LDH levels were detected. LDH amount was expressed as fold changes to the normal group.

### ELISA

The brain tissues were homogenized and centrifuged for detecting the pro-inflammatory factors. The protein levels of IL-6 (K4144-100), TNF-α (K1051-100), and IL-1β (K4795-100; all from Biovision Inc.) were analyzed by the corresponding ELISA kits according to the manufacturer's protocols. The absorbance values were detected at 450 nm wavelength.

### Real-time quantitative polymerase chain reaction (RT-qPCR)

The total RNA from brain tissues and cells were extracted by TRIzol® reagent (Invitrogen; Thermo Fisher Scientific, Inc.). RT-qPCR were carried out using Brilliant II SYBR Green qRT-PCR 1-Step Master Mix (600825; Agilent Technology Co., Ltd.) on MiniAmp™ Plus Thermal Cycler (A37835; Thermal Fisher Inc.). All primers were designed and synthesized by Fenghui Biotech Co., Ltd. GAPDH was used for internal reference. The relative expression of indicated RNAs were calculated using 2^−ΔΔCt^ approaches. The sequences of the primers were as follows: GSDMD-N forward, 5′-GAGTGTGGCCTAGAGCTGG-3′ and reverse, 5′-GGCTCAGTCCTGATAGCAGTG-3′; GSDMD-N forward, 5′-GTGTGTCAACCTGTCTATCAAGG-3′ and reverse 5′-CATGGCATCGTAGAAGTGGAAG-3′; caspase-1 p20 forward, 5′-CTGGACCGAGTGGTTCCCTCAAGT-3′ and reverse 5′-GCTCTGGGC AGGCAGCAAATTCTT-3′; pro-caspase-1 forward, 5′-CCGGGGATCCCTCTTCATTG-3′ and reverse 5′-ACCCTTTCAGTGGTTGGCAT-3′; ASC forward, 5′-CATGAACTGATCGACAGGATG-3′ and reverse, 5′-GGACCTCCTCCAAATGTTTC-3′; NLRP3 forward, 5′-CCATCGGCAAGACCAAGA-3′ and reverse, 5′-ACAGGCTCAGAATGCTCATC-3′; GAPDH forward, 5′-ATGGTGAAGGTCGGTGTGAA-3′ and reverse, 5′-GAGTGGAGTCATACTGGAAC-3′.

### Western blotting assay

The cellular protein was extracted by pre-chilled RIPA lysis buffer (R00101; Solarbio Technology Co., Ltd.). BCA Protein Assay Kit (ZY80815; Zeye Biotech Co., Ltd) was used to quantified the proteins. The protein (20 μg) was electrophoresed on 10% SDS-PAGE (BL522A; Biosharp Technology Co., Ltd.) for 2 h at 120 V, and then transferred onto PVDF membranes (Millipore) for 2 h at 200 mA. Subsequently, the membranes were blocked with 10% skim milk. Afterwards, the membranes were incubated with primary antibodies, including anti-caspase-1 p20 (1:200, AG-20B-0042-C100, whatman, England), anti-pro-caspase-1 (1:200, ab138483, abcam), anti-ASC (1:100, ab70627, abcam, England), anti-GSDMD (1:200; 66387-1-Ig, Proteintech, Wuhan, China), anti-NLRP3 (1:150, ab4207; abcam, England), and anti-GAPDH (1:2000, ABP50152, Amyjet, USA) at 4 °C overnight, and with the rabbit anti-horse IgG conjugated horseradish peroxidase secondary antibody (1:1000, A-AR11515, all from Abgent) at room temperature for 2 h. Finally, protein bands were pictured using ECL reagent on BioSpectrum Gel Imaging System (UVP, Inc.). GAPDH was used to normalize protein expression levels.

### Flow cytometry assay

The cells were stained by SYTOX (KFS148, Biolab, Beijing, China) and caspase-1 (Amyjet Scientific, Wuhan, China). Attune NxT Flow Cytometer and its supporting software (Thermo Fisher Inc.) was used for flow cytometry assay. 5 μl of SYTOX and anti-caspase-1 antibody were added to each well of the 6-well plate, and the cells were resuspended at the density of 1 × 106 ml. The results were detected using a flow cytometry.

### Luciferase reporter assay

The wild and mutant types 3′-UTR region of STAT1 luciferase reporter vectors were designed and synthesized by Guangzhou RiboBio Co., Ltd. Cite 1 of NLRP3 was the sequence of promoter regions 277–287; cite2 of NLRP3 was the sequence of promoter regions 1072–1082; cite2 of NLRP3 was the sequence of promoter regions 1238–1248. The cells were lysed to detect luciferase activities using the Luciferase Reporter Assay Kit (K801-200; BioVision Tech Co., Ltd.) after 48 h. The luciferase activity was normalized to Renilla luciferase activity.

### RNA pull-down

RNA pull-down assay was performed by MagCapture™ RNA Pull Down Assay Kit (297-77501; Whatman Co., Ltd.) following the manufacturer's protocol. Briefly, the cells were lysed and incubated with the biotinylated MALAT1 probe and its control probe. Streptavidin-labeled magnetic beads were resuspended and incubated with the probes (50 pmol) at 4 °C overnight. Next, the beads were eluted from the RNA–protein complex. The proteins were resolved in SDS-PAGE and detected by western blotting assay. The MALAT1 primers sequences were: sense, 5′-CCATCGGCAAGACCAAGA-3′; anti-sense, 5′-ACAGGCTCAGAATGCTCAT C-3′.

### RNA binding protein immunoprecipitation (RIP) assay

RIP assay was performed using the RNA Immunoprecipitation Kit (P0101; Geneseed) according to the manufacturer’s protocol. Briefly, cells were resuspended in PBS at the density of 1 × 10^7^, then lysed on ice and centrifuged, after which 100 μl of supernatant was incubated with magnetic beads conjugated with human anti-STAT1 antibody (3472-30T) and negative control normal mouse IgG (6402-05; both from Biovision Inc.). The magnetic beads were eluted and RNA complex was purified to obtain the RNA for RT-qPCR analysis.

### Chromatin immunoprecipitation (ChIP) assay

The ChIP assay was conducted using SimpleChIP® Enzymatic Chromatin IP Kit (Magnetic Beads) (cat no. 9003 s; Cell Signaling Technology Inc.) followed the manufacturer's protocol. Briefly, cells were resuspended. Then, cells were crosslinked with 1% formaldehyde at 37 °C for 30 min. 0.125 M glycine was added to stop the reaction. Cells were lysed on ice and ultrasonic-treated to obtain the genomic DNA fragments. Subsequently, the fragments were coprecipitated with anti-STAT1 antibody (3472-30T). The specific primers that were used to amplify the NLRP3 promoter DNA fragments. The DNA was purified for RT-qPCR analysis.

### Fluorescence in situ hybridization (FISH) assay

MALAT1 cellular expressions were analyzed using the Fluorescence In Situ Hybridization kit (F11201/50; Shanghai GenePharma Co., Ltd.) according to the manufacturer’s protocol. Briefly, cells were inoculated into a 48-well plate and the medium was discarded. The cells were rinsed with PBS twice and then fixed with 4% paraformaldehyde for 15 min. Thereafter, cells were hybridized with the Cyanine dye 5 (Cy5)-labeled MALAT1 (designed and synthesized by GenePharma) probes at 37 °C overnight. In the next day, the probe mixture was discarded and the cells were counterstained with DAPI. The cells were observed and captured under a fluorescence microscope.

### Statistical analysis

The data were analyzed using SPSS 20.0, and represented as mean ± SD. Student t test was applied for analyzing the difference between two groups, and one-way ANOVA for the difference among multiple groups. P < 0.05 was deemed as statistical significance.

## Results

### The pyroptosis of microglias was more remarkable in diabetic cerebral ischemia

**Fig. 1:**
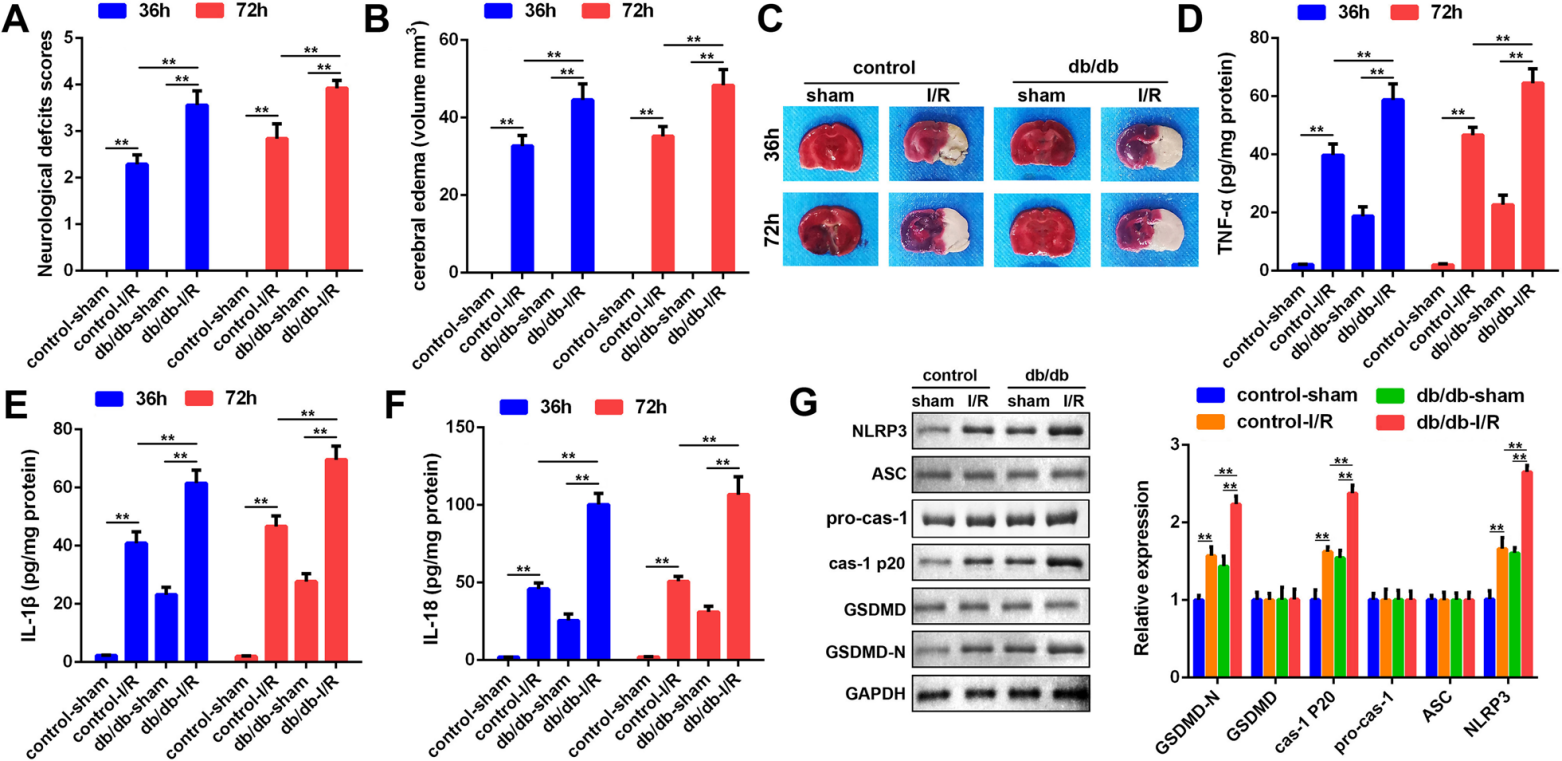
Diabetic mice suffered from worse cerebral injury relative to the control mice after cerebral I/R. **A** Neurological scores were assessed in the control and diabetic mice underwent I/R or sham operation. The assessment was performed 36 h (blue bar) and 72 h (red bar) after operation. **B** Encephaledema volume of the brain slices were calculated according to the formula: V = ∑(A1 + A2)t/2. V: volume of encephaledema (mm^3^); A1 and A2: front and back encephaledema area of the slides (mm^2^); t: thickness of the slides (mm). **C** The brain tissues were stained by TTC solution. Pale area represented for encephaledema. The concentrations of TNF-α (**D**), IL-1β (**E**), and IL-18 (**F**) in the brain tissues were measured by ELISA kit. **G** The protein expression (GSDMD-N, GSDMD, caspase-1 p20, pro-caspase-1, ASC and NLRP3) in the control-sham, control-I/R, db/db-sham and db/db-I/R groups were detected by western blot. ^**^P < 0.01, I/R, ischemia/reperfusion

The decrease of microglias was closely associated with the development of diabetic cerebral ischemia. As shown in Fig. 1A, the neurological deficit score of the I/R injury mice were significantly increased, which was more remarkable in db/db I/R group. The encephaledema volume of the I/R injury db/db mice was much larger than that of the I/R injury normal mice (Fig. 1B). This was consistent with the results from TTC staining. Diabetic cerebral ischemia further promoted brain tissue damage (Fig. 1C). Moreover, the levels of proinflammatory cytokines, including TNF-α, IL-18 and IL-1β were measured. As shown in Fig. 1D–F, the levels of the pro-inflammatory cytokines was significantly increased in I/R group, especially in db/db I/R mice. Previous studies revealed that inflammatory-induced microglial death (pyroptosis) is the key factor of cerebral I/R injury. We, thereafter, determined the expressions of pyroptosis biomarkers, NLRP3, ASC, caspase-1, and GSDMD. The protein expression of NLRP3, caspase-1 p20, and GSDMD-N was significantly increased in I/R group, which more potent in diabetic cerebral ischemia mice (Fig. 1G).

### Knockdown of MALAT1 relieved cerebral I/R injury in diabetic mice

**Fig. 2:**
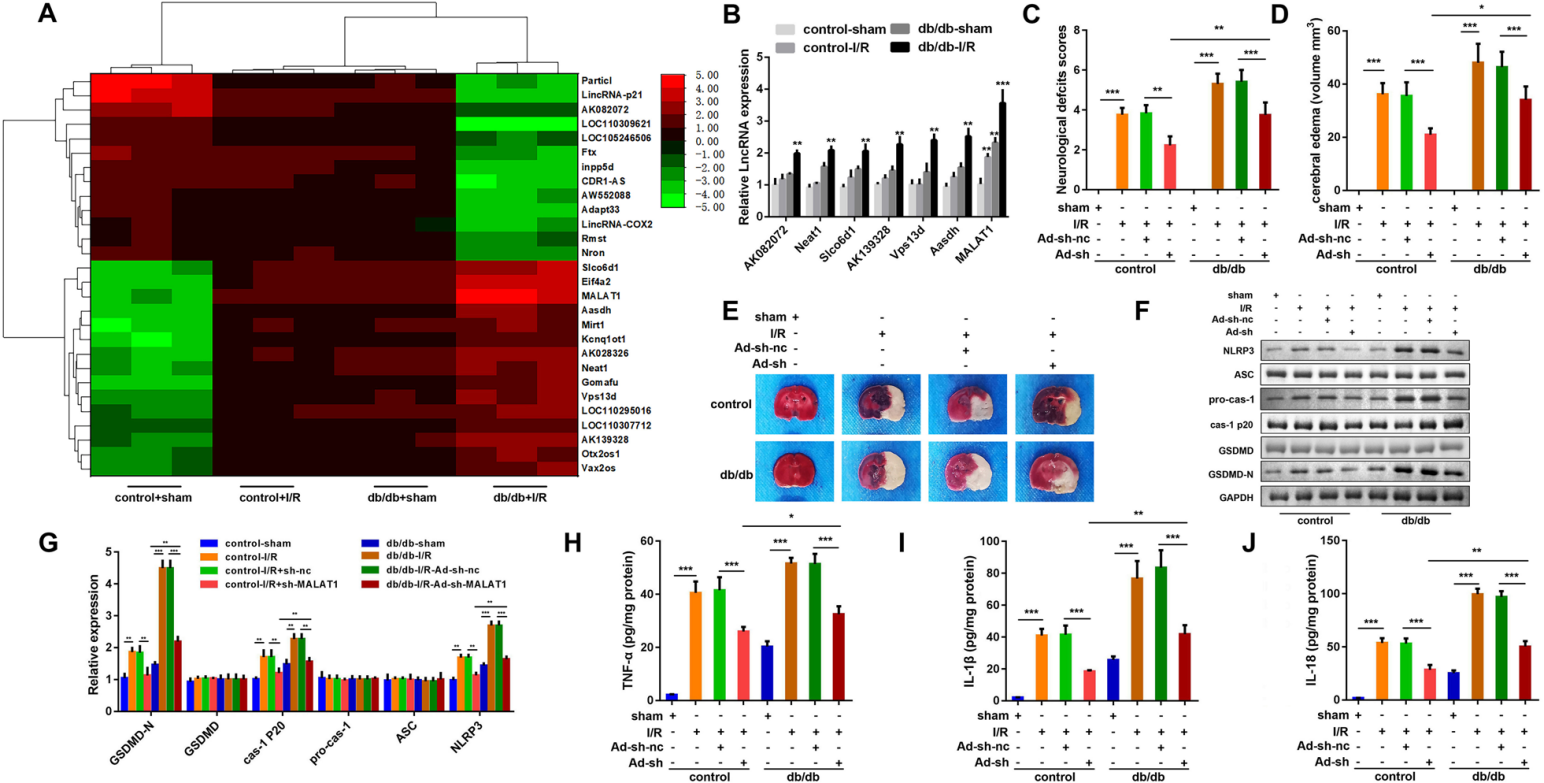
Knockdown of MALAT1 ameliorated cerebral injury and pyroptosis of the diabetic I/R mice. **A** The aberrant expressed lncRNAs in diabetic cerebral ischemia screened by bioinformatic analysis. **B** The expression of lncRNAs in mouse brain tissues. **C** Neurological scores were assessed in the diabetic mice underwent I/R or sham operation. Adenovirus harboring MALAT-1 short hairpin RNA (ad-sh-MALAT1) was infected into the diabetic I/R mice through intraventricular injection. The assessment was performed 72 h after operation. **D** The brain tissues were isolated from the treated mice and sliced into 2 mm thickness after they were euthanasia. Encephaledema volume of the slides were calculated according to the formula: V = ∑(A1 + A2)t/2. V: volume of encephaledema (mm^3^); A1 and A2: front and back encephaledema area of the slides (mm^2^); t: thickness of the slides (mm). **E** The brain tissues were stained by TTC solution. Pale area represented for encephaledema. **F**, **G** The protein expression of GSDMD-N, GSDMD, caspase-1 p20, pro-caspase-1, ASC and NLRP3 were separated by western blotting assay. The concentrations of TNF-α (**H**), IL-18 (**I**), and IL-1β (**J**) in the brain tissues were measured by ELISA kit. (n = 6), ^**^P < 0.01, I/R, ischemia/reperfusion

Through bioinformatic approaches, we found that several lncRNAs were aberrant expressed in diabetic cerebral ischemia (Fig. 2A). We screened for seven upregulated lncRNAs, among which MALAT1 was most significantly upregulated (Fig. 2B). Therefore, MALAT1 was selected for the following study. MALAT1 knockdown efficiently alleviated neurological deficit, increased encephaledema volume, and suppressed diabetic cerebral ischemia induced brain tissue damage (Fig. 2C–E). Moreover, knockdown of MALAT1 significantly reduced the expression of GSDMD-N, capase-1 p20 and NLRP3 induced by cerebral I/R injury (Fig. 2F an G). Moreover, MALAT1 knockdown significantly decreased TNF-α, IL-18 and IL-1β levels (Fig. 2H–J).

### High glucose facilitated the pyroptosis of microglias induced by H/R in vitro

**Fig. 3:**
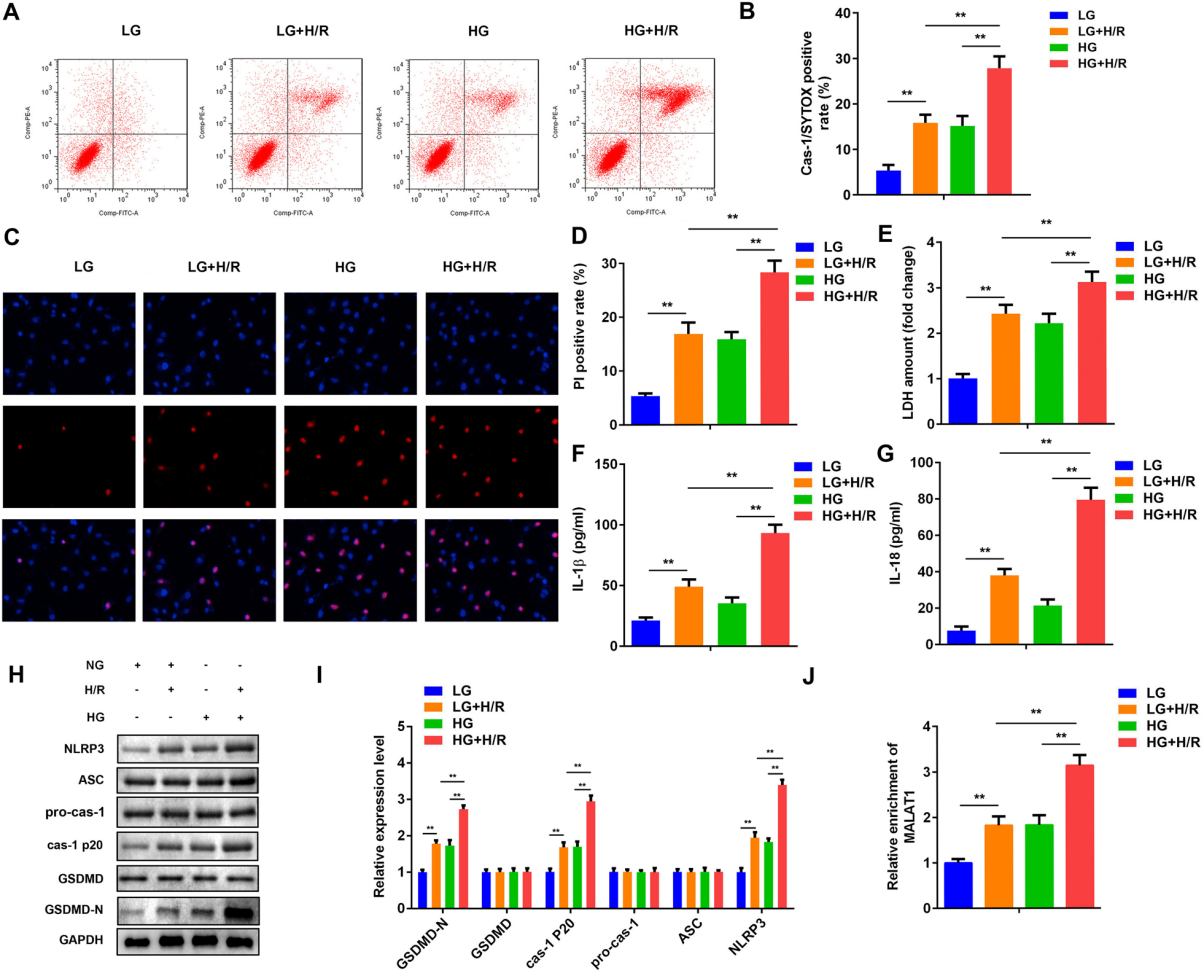
High glucose treatment aggravated cytotoxicity and pyroptosis of the H/R BV2 cells. **A**, **B** 72 h after the indicated treatment, BV2 cells were labelled with caspase-1 antibody and SYTOX, and counted by flow cytometry. **C**, **D** The cells were stained by PI and DAPI 72 h after the indicated treatment. PI positive rate was expressed as the proportion of PI positive cells (red) in the DAPI positive cells (blue). **E** LDH concentration of the BV2 cells were measured by ELISA kit 72 h after indicated treatment. The concentrations of IL-18 (**F**), and IL-1β (**G**) in the BV2 cells were measured by ELISA kit. **H**, **I** Western blot for GSDMD-N, GSDMD, caspase-1 p20, pro-caspase-1, ASC and NLRP3 of the BV2 cells 72 h after they received indicated treatment. The optical density (OD) of protein bands was indicated relative to corresponding loading control GAPDH. **J** MALAT1 levels were measured by RT-qPCR 72 h after the indicated treatment. (n = 6), ^**^P < 0.01, OGD/R, oxygen–glucose deprivation/reoxygenation

Cells were exposed to high glucose (HG) to mimic microglial cells in diabetic mice, and H/R was used to mimic I/R injury in vitro. As shown in Fig. 3A–D, H/R significantly increased cas-1/SYTOX positive cells as well as PI positive cells. Moreover, the increase of LDH level was further enhanced by HG (Fig. 3E). Additionally, HG increased the release of pro-inflammatory cytokyines, such as IL-18 and IL-1β (Fig. 3F and G). GSDMD-N, caspase-1 p20 and NLRP3 expressions were facilitated in the H/R cells. HG significantly increased the expression of GSDMD-N, caspase-1 p20 and NLRP3 of H/R-treated cells. Moreover, H/R treatment significantly increased the concentration of IL-18 and IL-1β, which was more potent in HG + H/R group (Fig. 3H and I). HG facilitated the expression of MALAT1 (Fig. 3J).

### Downregulating MALAT1 mitigated pyroptosis of microglias induced by diabetic cerebral ischemia in vitro

**Fig. 4:**
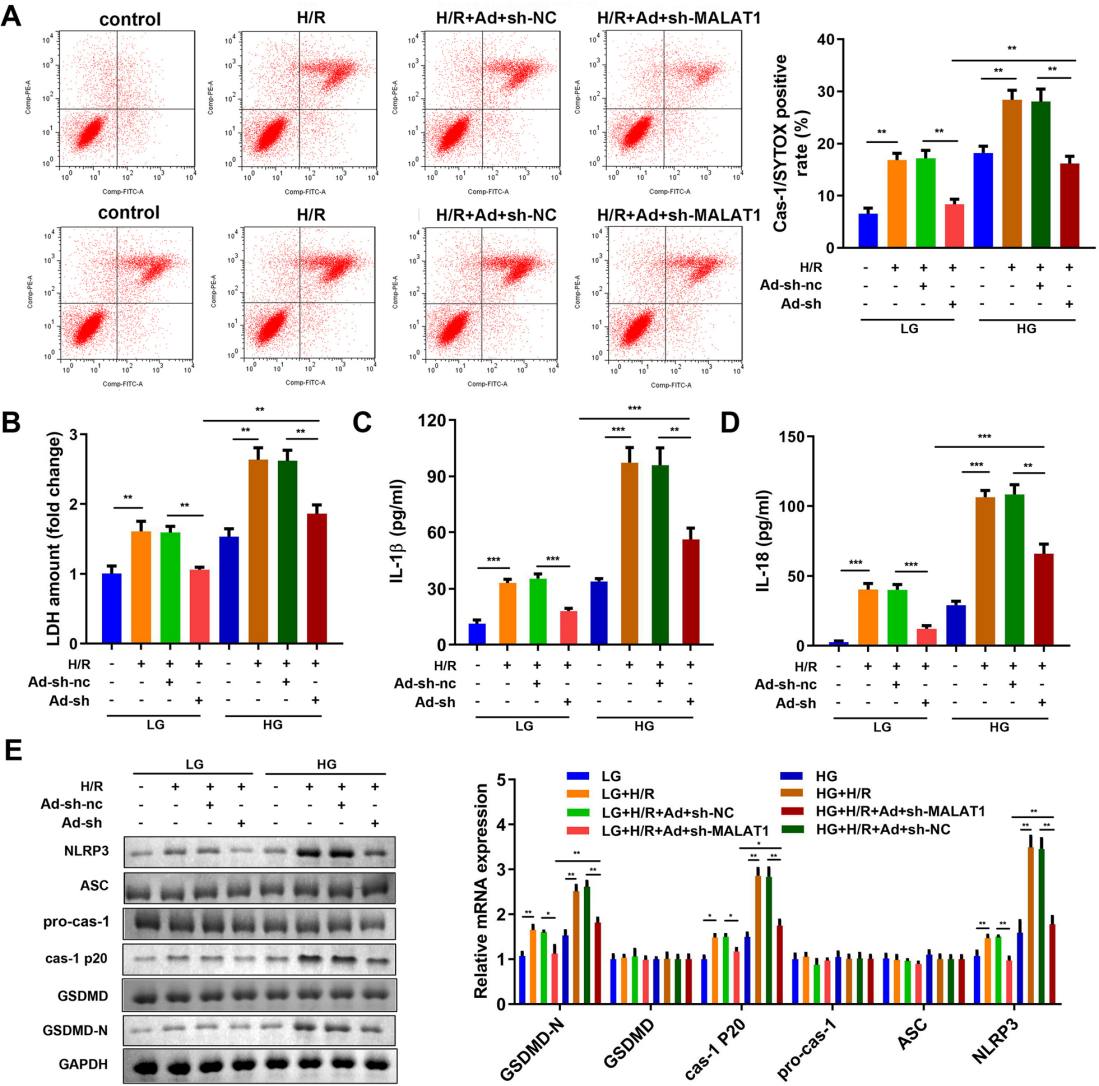
Downregulation of MALAT1 inhibited pyroptosis and cytotoxicity induced by high glucose and H/R of the BV2 cells. **A** Cspase-1/SYTOX positive cells were counted by flow cytometry. **B** LDH concentrations of the BV2 cells were detected with ELISA kit 72 h after indicated treatments. LDH amount was expressed as the fold to the LDH level of the NG group. IL-1β (**C**) and IL-18 (**D**) and concentrations in the BV2 cells were measured by ELISA kit 72 h after indicated treatments. **E** The protein expression of GSDMD-N, GSDMD, caspase-1 p20, pro-caspase-1, ASC and NLRP3 were separated by western blotting assay. (n = 6), ^**^P < 0.01, H/R, hypoxia/reoxygenation; LDH, lactate dehydrogenase

To futher verify the potential roles of MALAT1 in diabetic cerebral ischemia, we examined the effects of MALAT1 on diabetic cerebral ischemia model in vitro. The transfection efficiency of sh-MALAT1 was showed in Additional file 1: Fig. S1. As shown in Fig. 4A, MALAT1 knockdown significantly decreased cas-1/SYTOX positive cells. Moreover, downregulation of MALAT1 significantly suppressed cytotoxicity (Fig. 4B) and inflammatory response (Fig. 4C and D), manifested by the decrease in the release of LDH, IL-18 and IL-1β. Moreover, the protein expression levels of GSDMD-N, caspase-1 p20 and NLRP3 elevated by HG and H/R treatments was antagonized by MALAT1 knockdown (Fig. 4E).

### MALAT1 positively regulated NLRP3 expression via binding to STAT1

**Fig. 5:**
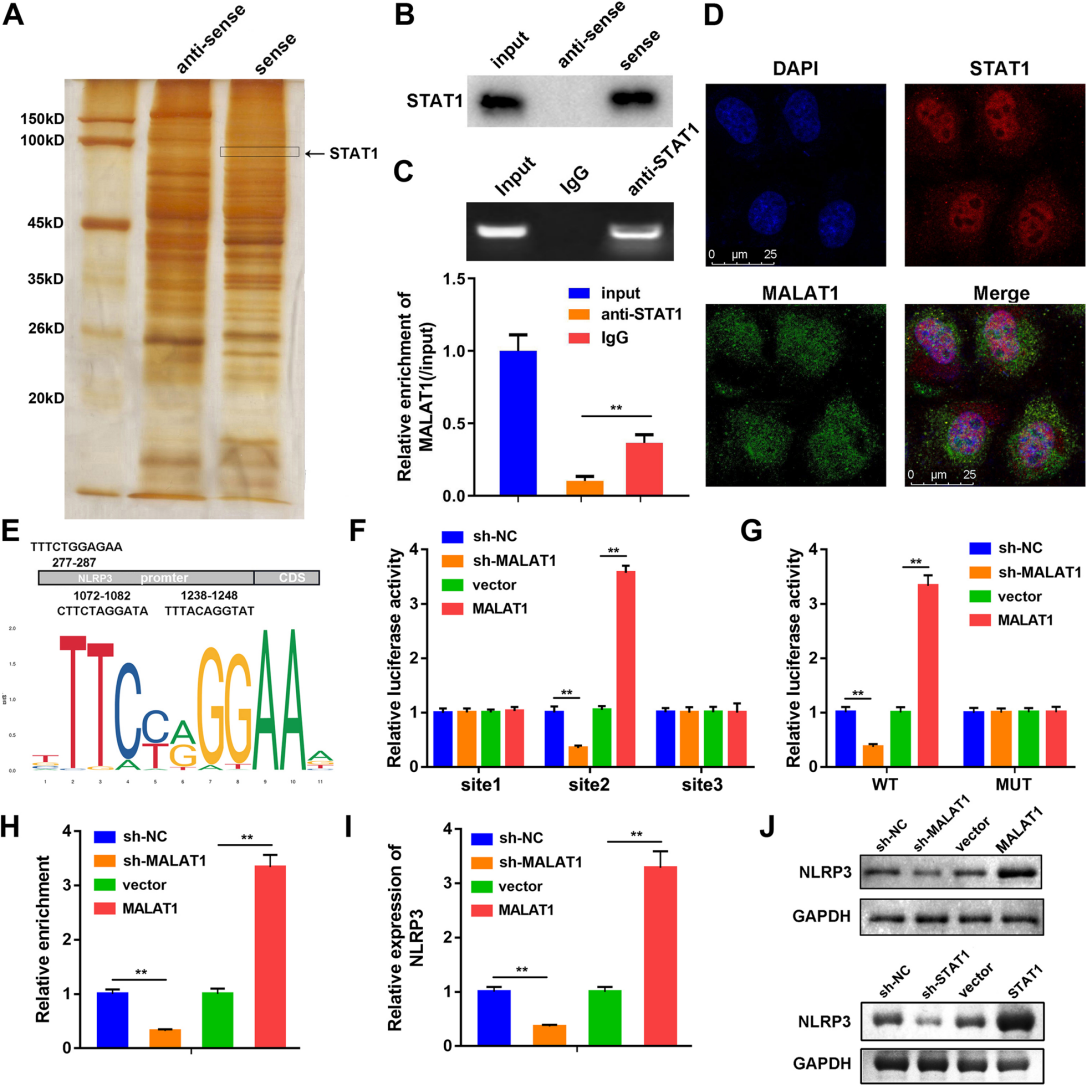
MALAT1 positively regulated NLRP3 expression level through binding to STAT1. **A** The protein bands of sense and anti-sense MALAT1 groups after RNA-pull down assay was performed. STAT1 was marked in the image. **B** RNA pull-down assay for STAT1 in input, anti-sense and sense MALAT1 groups. **C** MALAT1 enrichment of input, IgG and anti-STAT1 groups were detected by RIP assay. The MALAT1 relative enrichment was normalized on input. **D** FISH result exhibited the subcellular localization of MALAT1 (red) and STAT1 (green) of the BV2 cells. The cells were hybridized with the Cy-2 labeled anti-STAT1 and Cy-7 labeled MALAT1 probes. **E** The potential binding site sequences of STAT1 with NLRP3 (upper). DNA binding motif of STAT1 predicted by Jaspar (lower). **F** Relative luciferase activities of the BV2 cells were detected 48 h after transfection of luciferase reporter vectors. **G** Relative luciferase activities of the BV2 cells were detected 48 h after they were transfected with wild and mutant types STAT1 luciferase reporter vectors. **H** The affinity of STAT1 in the promoter of NLRP3 detected using ChIP assay. **I** NLRP3 levels in the the BV2 cells were measured by RT-qPCR after MALAT1 knockdown or overexpression. **J** Western blot for NLRP3 of the BV2 cells transfected with indicated ad-shRNA or overexpression plasmids. (n=3), ^**^P<0.01, NC, negative control. Cite 1, the sequence of NLRP3 promoter regions 277–287; cite2, the sequence of NLRP3 promoter regions 1072–1082; cite2, the sequence of NLRP3 promoter regions 1238–1248

Our preliminary experiment used Genome (http://genome.ucsc.edu/) to locate the promoter region of MALAT1. And ALGGEN (http://alggen.lsi.upc.es/) predicted that STAT1, a transcription factor, was able to bind to the promoter. We carried out RNA-pull down assay for validation. Anti-sense MALAT1 was used as a negative control. RNA pull-down (Fig. 5A and B) and mass spectrometry analysis (Additional file 4: material S1) showed MALAT1 could interact with STAT1. Next, RIP assay further confirmed the interaction between MALAT1 and STAT1 (Fig. 5C). In the HG treated cells, the interaction between MALAT1 and STAT1 was also confirmed (Additional file 2: Fig. S2). Cellular colocalization images showed that MALAT1 and STAT1 were mainly expressed in the nuclear, suggesting that MALAT1 may induce pyroptosis via interacting with STAT1 (Fig. 5D). Subsequently, the results of Genome and ALGGEN showed that STAT1 had binding sites with the promoter sequence of NLRP3. The sequences were predicted and proved by JASPAR (http://jaspar.genereg.net/) (Fig. 5E). This was further verified by luciferase activity. As shown in Fig. 5F, knockdown of MALAT1 significantly decreased the luciferase activity in microglias (Fig. 5G). ChIP assay showed that STAT1 had high affinity in promoter region of NLRP3 (Fig. 5H). The mRNA and protein expression of NLRP3 was positively regulated by MALAT1 and STAT1 (Fig. 5I, J).

### STAT1 knockdown alleviated MALAT1-promoted pyroptosis of microglias

**Fig. 6:**
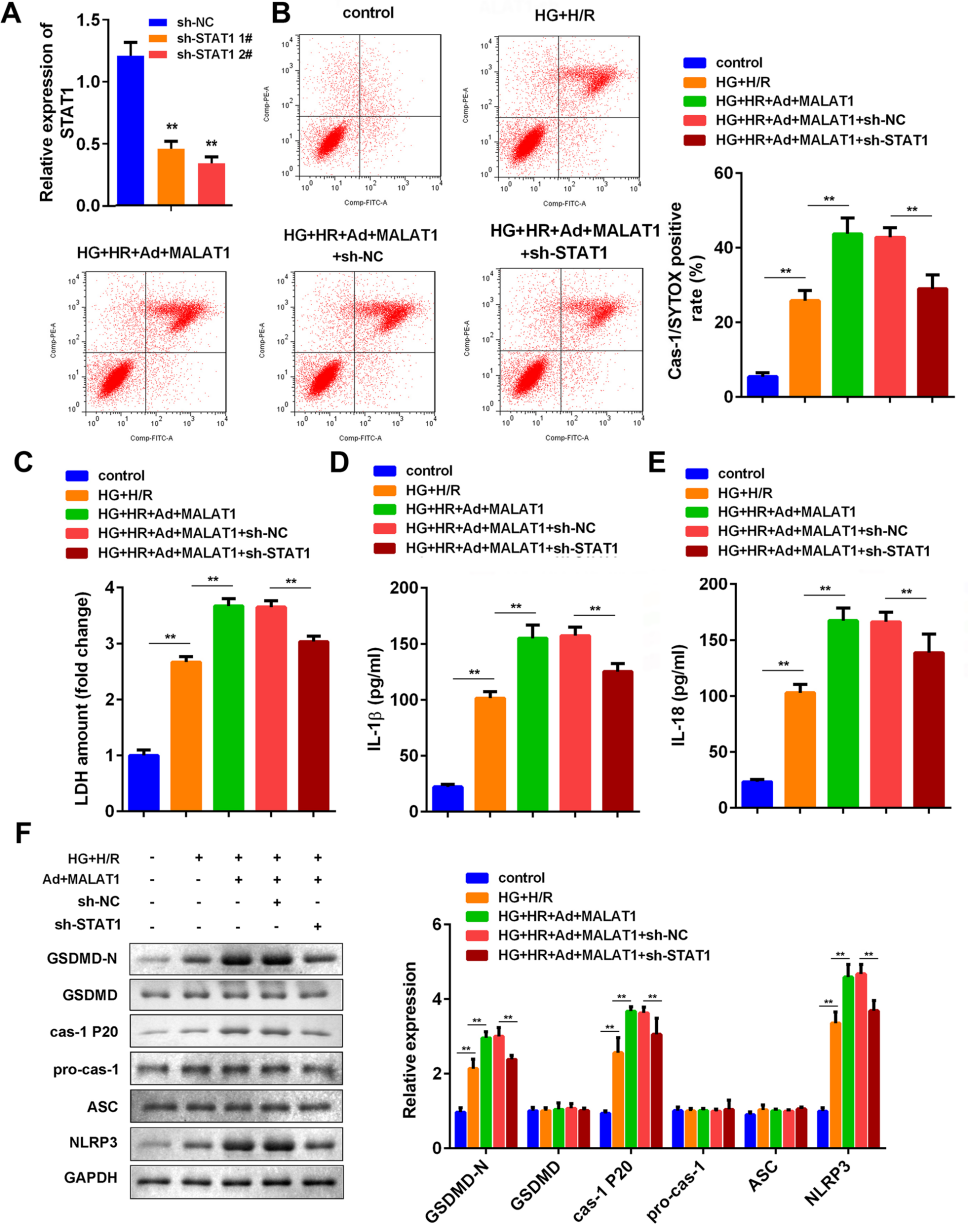
Knockdown of STAT1 reversed the effect of MALAT1 on pyroptosis induced by high glucose and H/R in BV2 cells. **A** The mRNA levels of STAT1 in the BV2 cells transfected with indicated ad-shRNA. **B** The cells were stained by SYTOX and cas-1. **C** LDH levels were measured by ELISA kit of the BV2 cells 72 h after indicated treatment. IL-1β (**D**) and IL-18 (**E**) concentrations of the BV2 cells were measured by ELISA kits 72 h after indicated treatments. **F** Western blot for GSDMD-N, GSDMD, caspase-1 p20, pro-caspase-1, ASC and NLRP3 of the BV2 cells 72 h after indicated treatment. (n = 6), ^**^P < 0.01, H/R, hypoxia/reoxygenation; LDH, lactate dehydrogenase; NC, negative control

Considering si-STAT1 inhibited NLRP3 protein expression, we then investigated the effects of STAT1 knockdown on pyroptosis of microglias. STAT1 expression level was notably reduced by sh-STAT1 1# and 2# (Fig. 6A). Then sh-STAT1 1# was applied in the following experiments. SYTOX/caspase1-positive rates was significantly enhanced by MALAT1 overexpression, while STAT1 knockdown reversed this effect of MALAT1 (Fig. 6B). Also, knockdown of STAT1 reversed the effect of MALAT1 overexpression on the cytotoxicity (Fig. 6C). In addition, STAT1 silencing significantly reduced IL-18 and IL-1β concentrations compared to the MALAT1 overexpression group (Fig. 6D and E). As we expected, the protein expression of GSDMD-N, caspase-1 p20 and NLRP3 were elevated by MALAT1 overexpression, but reversed by STAT1 knockdown (Fig. 6F). Additionally, we found that knockdown of STAT1 alone in HG + HR treated cells showed the same effect as in HG + HR treated cells transfected with MALAT1 (Additional file 3: Fig. S3).

### Knockdown of STAT1 reversed the effects of MALAT1 on cerebral injury of the diabetic I/R mice

**Fig. 7:**
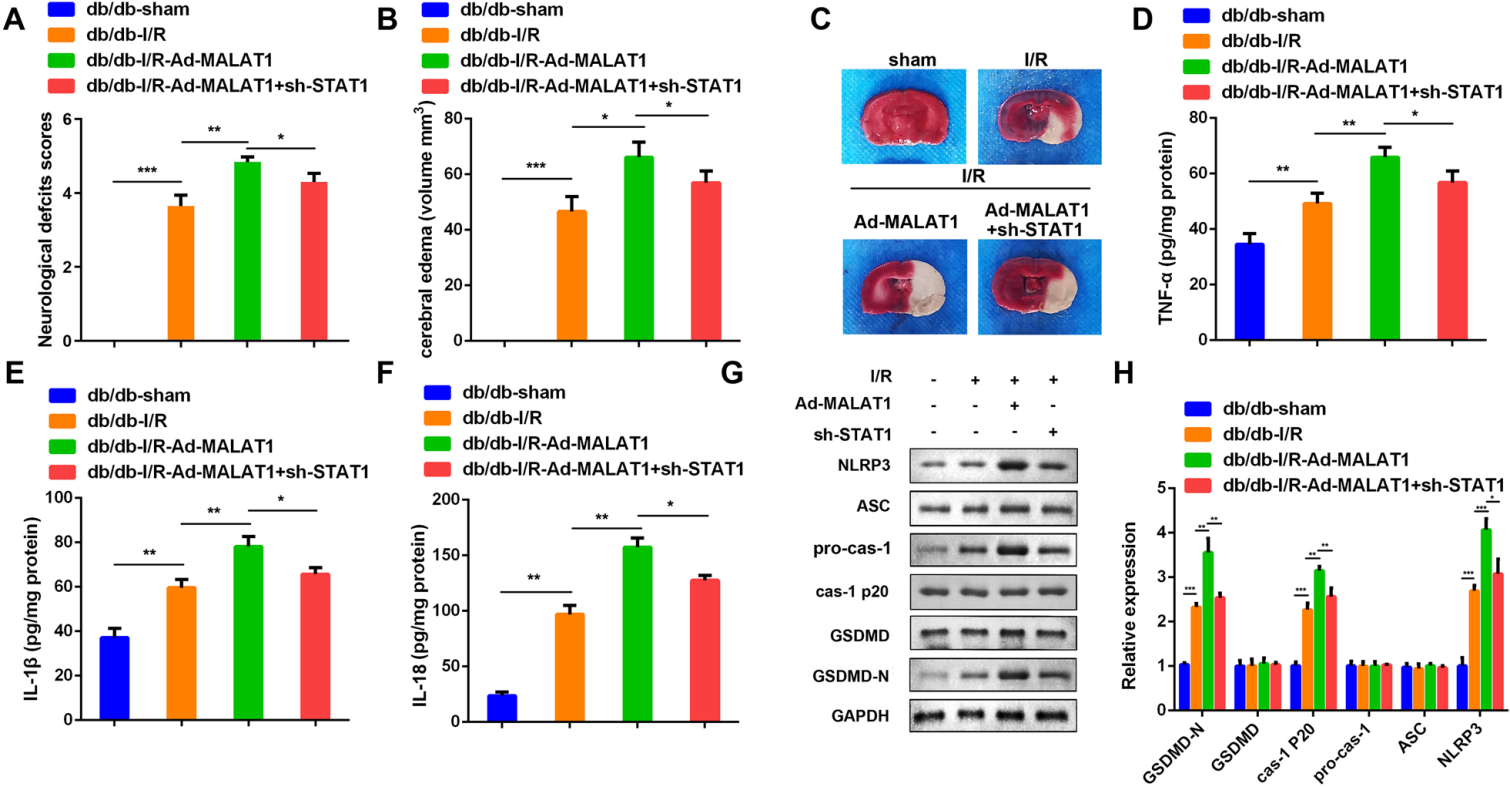
Knockdown of STAT1 reversed the effect of MALAT1 on cerebral injury and pyroptosis of the diabetic I/R mice. **A** Neurological scores were assessed in vivo. **B** Encephaledema volume of the brain slices were calculated according to the formula: V = ∑(A1 + A2)t/2. V: volume of encephaledema (mm^3^); A1 and A2: front and back encephaledema area of the slides (mm^2^); t: thickness of the slides (mm). **C** The brain slices were stained by TTC solution. Pale area represented for encephaledema. The concentrations of TNF-α (**D**), IL-1β (**E**) and IL-18 (**F**) in the brain tissues were measured by ELISA kit. **G**, **H** The protein expression levels of GSDMD-N, GSDMD, caspase-1 p20, pro-caspase-1, ASC and NLRP3 in the mice underwent indicated treatment were detected by RT-qPCR 72 h after I/R modeling. (n = 6), ^**^P < 0.01, I/R, ischemia/reperfusion

Ad-sh-STAT1 and Ad-MALAT1 were injected into the diabetic I/R mice to further investigate the effects of STAT1 knockdown and MALAT1 overexpression on diabetic cerebral ischemia in vivo. MALAT1 overexpression increased the neurological deficit scores in diabetic I/R mice, but STAT1 silencing decreased it compared to the MALAT1 overexpression group (Fig. 7A). Besides, injection of Ad-sh-STAT1 reduced the volume of encephaledema and suppressed brain tissues damages which could be enhanced by MALAT1 overexpression (Fig. 7B and C). Moreover, MALAT1 overexpression promoted the release of TNF-α, IL-18, and IL-1β, while STAT1 knockdown reversed this effect (Fig. 7D–F). The protein expression of pyroptosis biomarkers, including GSDMD-N, caspase-1 p20 and NLRP3, were significantly promoted by MALAT1 overexpression, and again, downregulated by STAT1 knockdown (Fig. 7G and H).

### MALAT1 expression level was regulated by STAT1

**Fig. 8:**
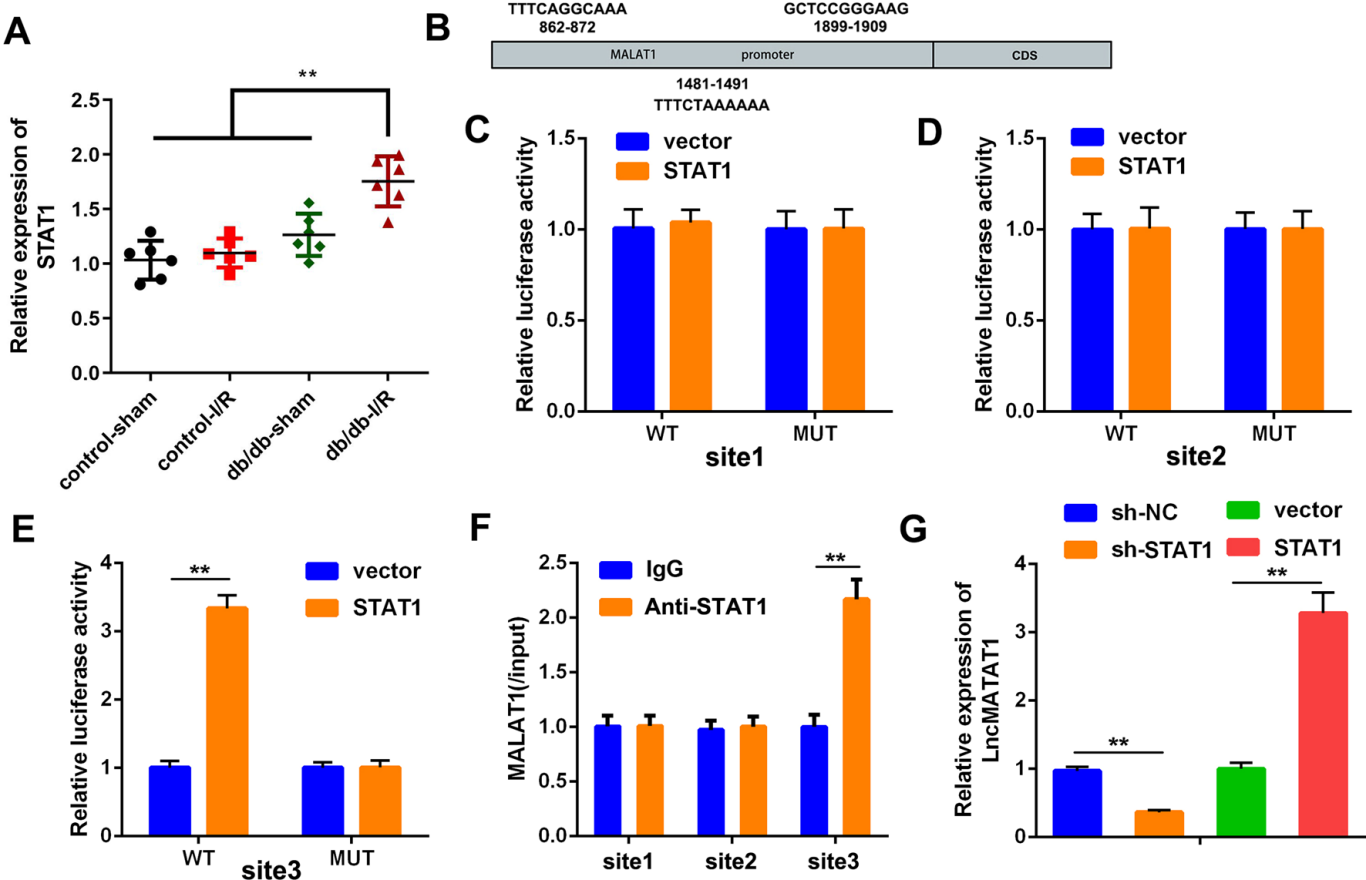
STAT1 targeted MALAT1 to promote its transcription. **A** STAT1 expression levels of the mice were assessed by RT-qPCR 72 h after the indicated treatments. **B** The binding sites between MALAT1 and STAT1 was showed. **C**–**E** The luciferase activities of the BV2 cells 24 h after transfection of the luciferase reporter vectors. Sequences of three STAT1 sites were cloned into the vectors, respectively. **F** MALAT1 enrichments of the three STAT1 sites were detected by RIP assay. The MALAT1 relative enrichment was normalized to input. **G** MALAT1 expression levels of the BV2 cells 72 h after the indicated treatment. (n = 3), ^**^P < 0.01

We had elucidated that MALAT1 promoted the pyroptosis of microglias via binding to STAT1. Interestingly, we found that the promoter of MALAT1 has the potential binding sites of STAT1. Thus, we tried to figure out whether STAT1 could regulate the transcription of MALAT1. We found that the mRNA expression of STAT1 was significantly increased in I/R + db/db mice (Fig. 8A). STAT1 was predicted to have three complementary targeting sites in the promoter region of MALAT1 (Fig. 8B). Moreover, luciferase assay indicated that STAT1 remarkable increased the luciferase activity in site 3, whereas there was no significant changes in site 1 and 2, which means STAT1 could bind with the site 3 of the MALAT1 promoter (Fig. 8C–E). RIP data showed that the MALAT1 enrichment of STAT1 was notably higher than that of IgG in site 3 (Fig. 8F). These data suggested that STAT1 targeted MALAT1 on site 3. Moreover, MALAT1 transcription level was significantly reduced by STAT1 knockdown, but increased by STAT1 overexpression (Fig. 8G).

## Discussion

In this study, we demonstrated that knockdown of MALAT1 relieved diabetic cerebral ischemia for the first time. MALAT1 was upregulated in diabetic cerebral ischemia. However, knockdown of MALAT1 improved neurological deficits of the diabetic I/R mice model and ameliorated pyroptosis of microglias. Mechanically, we found that MALAT1 interacted with the transcription factor STAT1 to induce the activation of NLRP3 inflammasome. Knockdown of MALAT1 inactivated NLRP3 signaling and suppressed the pyroptosis of microglias and brain tissue damage induced by diabetic cerebral ischemia via interacting with STAT1. Moreover, STAT1 transcriptionally activated MALAT1. Hence, MALAT1 interacting with STAT1 promoted diabetic cerebral ischemia-induced pyroptosis through regulating NLRP3 signaling.

NLRP3 inflammasome, activated by various microbe-associated molecular patterns and damage-associated molecular patterns (DAMPs) induces the cascade of inflammatory response and damages brain tissues (Brennan and Cookson 2000; Frank and Vince 2019). Post-transcriptional NLRP3 has been shown to elicit cellular inflammatory responses, leading to a range of validation-related diseases (Raimondo et al. 2013). Pyroptosis, a pro-inflammatory pattern of death stimulated by inflammasome activation, is closely associated with microglia cell dysfunction and brain tissue damage. Pyroptosis is different from necrosis: (1) the activation of NLRP3 inflammasome (Yu et al. 2021; Xu et al. 2020; Tu et al. 2019); (2) GSDMD mediated rupturing and blebbing of plasma membrane, which is the characteristics of pyroptosis (Wang et al. 2020); (3) with canonical inflammasomes (engaging pro-caspase-1) and non-canonical inflammasomes (activating caspase-11) (He et al. 2015). Diabetic cerebral ischemia is an autoimmune disease (Zhang et al. 2019). Diabetes mellitus suppresses white matter repair and promotes long-term cerebral ischemia injuries (Ma et al. 2018). Diabetic cerebral ischemia stimulated NLRP3 inflammasomes (NLRP3, ASC and cas-1) and induced microglia cell dysfunction (inflammatory response and pyrotosis). Thence, to suppress the activation of NLRP3 may be a potential strategy for diabetic cerebral ischemia injuries.

LncRNAs are widely employed as targeting treatments for several diseases with the advantages of high specificity and mild side effects. It promoted high glucose-induced H9C2 cardiomyocyte pyroptosis via targeting miR-141-3p (Wu et al. 2021). A previous study also revealed that MALAT1 accelerated high glucose-induced pyroptosis of endothelial cells partly by upregulating NLRP3 expression. Wang et al. (2018) demonstrated that MALAT1 mediated the exacerbation of cerebral I/R injury induced by diabetes through triggering the inflammatory response in microglia via MyD88 signaling. Therefore, we hypothesized that knockdown of MALAT1 might relieve diabetic cerebral ischemia by inhibiting pyroptosis. We found that knockdown of MALAT1 ameliorated neurological deficits, encephaledema, and pyroptosis in vivo and restored microglia cell function in vitro. MALAT1 knockdown mediated inactivation of NLRP3 may protected against diabetic cerebral ischemia.

LncRNAs function in various pathological processes of human body through different mechanism, one of which is binding to specific proteins. lncRNAs function as ceRNA to regulate gene expression via sponging microRNAs. lncRNAs also interacts with RNA binding protein to promote the mRNA stability of the target gene. Additionally, lncRNAs bind to transcription factors to transcriptionally regulate gene expression. For instance, Ni et al. reveal that lncRNA GAS5 induces YAP phosphorylation, which further downregulates YTHDF3 suppresses m6A motification of GAS5 (Ni et al. 2019). FOXN3-NEAT1-SIN3A forms a negative feedback loop to exacerbate the carcinogenesis of hormonally responsive breast cancer (Li et al. 2017b). lncRNA uc.134 inhibits the aggressiveness of hepatocellular carcinoma via suppressing CUL4A-mediated ubiquitination of LATS1 (Ni et al. 2017). In this study, lncRNA MALAT1 bound with STAT1 to induce the upregulation of NLRP3. STAT families determines immune responses in the microenvironment in tumor as well as in nerve disorders (Yu et al. 2009; Butturini et al. 2019). STAT1-mediated downregulation of *N*-methyl-d-aspartate receptors contributes to hippocampal neuron degeneration and memory deficits (Li et al. 2019). Inactivation of JAK/STAT1 signaling and IL-13 suppresses the pyroptosis and alleviates moderate traumatic brain injury (Gao et al. 2020). In this study, STAT1 bound to the promoter region of NLRP3 and induce the inflammatory response in microglias, which finally increased the accumulation of pyrotosis executor GSDMD-N. Interestingly, MALAT1 knockdown suppressed the transcriptional activity of STAT1 and restored microglia cellular function. This may provide a new sight of diabetic cerebral ischemia.

We subsequently attempted to investigate the upstream regulatory mechanism of MALAT1 after we had demonstrated the MALAT1/STAT1/NLRP3 pathway. Interestingly, STAT1 was predicted to have three binding sites with the promoter of MALAT1, and we proved that STAT1 regulated the transcription of MALAT1 via targeting the promoter of it.

## Conclusion

In conclusion, knockdown of lncRNA MALAT1 suppresses pyroptosis through inhibiting NLRP3 expression via STAT1, and MALAT1 transcription is regulated by STAT1 during diabetic cerebral ischemia. This study might contribute to a better understanding of diabetic cerebral ischemia pathogenesis and provide a basis for developing novel therapeutic strategies.

## Supplementary Information

**Additional file 1: Figure 1.** Verification of transfection efficiency of sh-MALAT1. The MALAT1 levels were detected by RT-qPCR after sh-MALAT 1# and sh-MALAT 2# transfection. ^**^P<0.01.

**Additional file 2: Figure 2.** Verification of the interaction between MALAT1 and STAT1 in the HG treated cells. In the HG treated cells, the interaction between MALAT1 and STAT1 was also confirmed by RNA pull down assay.

**Additional file 3: Figure 3.** Knockdown of STAT1 decreased pyroptosis rate in high glucose and H/R treated BV2 cells. (A) after the indicated treatment, BV2 cells were labelled with caspase-1 antibody and SYTOX, and counted by flow cytometry.(B) LDH concentration of the BV2 cells were measured by ELISA kit after indicated treatment. The concentrations of IL-18 (C), and IL-1β (D) in the BV2 cells were measured by ELISA kit. (E) Western blot for GSDMD-N, GSDMD, caspase-1 p20, pro-caspase-1, ASC and NLRP3 of the BV2 cells 72 h after they received indicated treatment. (n = 6), ^*^P<0.05, ^**^P<0.01.

**Additional file 4.** Mass spectrometry analysis results.

## Data Availability

The datasets used and/or analyzed during the current study are available from the corresponding author on reasonable request.

## Abbreviations

CCA: Common carotid artery
DN: Diabetic nephropathy
ECA: External carotid artery
GSDMD: Gasdermin D
ICA: Internal carotid artery
LDH: Lactate dehydrogenase
si-MALAT1: MALAT1 small interfering RNA
MALAT1: Metastasis Associated Lung Adenocarcinoma Transcript 1
MCAO: Middle cerebral artery occlusion
MCAO: Middle cerebral artery occlusion
NLRP3: Nucleotide-binding oligomerization domain 3
PRRs: Pattern recognition receptors
STAT1: Signal transducer and activator of transcription-1
TTC: Triphenyltetrazolium chloride

## References

[CR1] Brennan MA, Cookson BT (2000). Salmonella induces macrophage death by caspase-1-dependent necrosis. Mol Microbiol.

[CR2] Butturini E, Boriero D, Carcereri de Prati A, Mariotto S (2019). STAT1 drives M1 microglia activation and neuroinflammation under hypoxia. Arch Biochem Biophys..

[CR3] Della Corte V, Tuttolomondo A, Pecoraro R, Di Raimondo D, Vassallo V, Pinto A (2016). Inflammation, endothelial dysfunction and arterial stiffness as therapeutic targets in cardiovascular medicine. Curr Pharm Des.

[CR4] Di Raimondo D, Tuttolomondo A, Buttà C, Casuccio A, Giarrusso L, Miceli G, Licata G, Pinto A (2013). Metabolic and anti-inflammatory effects of a home-based programme of aerobic physical exercise. Int J Clin Pract.

[CR5] Ding C, He Q, Li PA (2005). Diabetes increases expression of ICAM after a brief period of cerebral ischemia. J Neuroimmunol.

[CR6] Frank D, Vince JE (2019). Pyroptosis versus necroptosis: similarities, differences, and crosstalk. Cell Death Differ.

[CR7] Gao C, Yan Y, Chen G, Wang T, Luo C, Zhang M, Chen X, Tao L (2020). Autophagy activation represses pyroptosis through the IL-13 and JAK1/STAT1 pathways in a mouse model of moderate traumatic brain injury. ACS Chem Neurosci.

[CR8] Han Y, Qiu H, Pei X, Fan Y, Tian H, Geng J (2018). Low-dose sinapic acid abates the pyroptosis of macrophages by downregulation of lncRNA-MALAT1 in rats with diabetic atherosclerosis. J Cardiovasc Pharmacol.

[CR9] He WT, Wan H, Hu L, Chen P, Wang X, Huang Z, Yang ZH, Zhong CQ, Han J (2015). Gasdermin D is an executor of pyroptosis and required for interleukin-1β secretion. Cell Res.

[CR10] Li ZG, Britton M, Sima AA, Dunbar JC (2004). Diabetes enhances apoptosis induced by cerebral ischemia. Life Sci.

[CR11] Li X, Zeng L, Cao C, Lu C, Lian W, Han J (2017). Long noncoding RNA MALAT1 regulates renal tubular epithelial pyroptosis by modulated miR-23c targeting of ELAVL1 in diabetic nephropathy. Exp Cell Res.

[CR12] Li W, Zhang Z, Liu X, Cheng X, Zhang Y, Han X, Zhang Y, Liu S, Yang J, Xu B, He L, Sun L, Liang J, Shang Y (2017). The FOXN3-NEAT1-SIN3A repressor complex promotes progression of hormonally responsive breast cancer. J Clin Invest.

[CR13] Li XG, Hong XY, Wang YL, Zhang SJ, Zhang JF, Li XC, Liu YC, Sun DS, Feng Q, Ye JW, Gao Y, Ke D, Wang Q, Li HL, Ye K, Liu GP, Wang JZ (2019). Tau accumulation triggers STAT1-dependent memory deficits by suppressing NMDA receptor expression. EMBO Rep.

[CR14] Liu C, Zhuo H, Ye MY, Huang GX, Fan M, Huang XZ (2020). LncRNA MALAT1 promoted high glucose-induced pyroptosis of renal tubular epithelial cell by sponging miR-30c targeting for NLRP3. Kaohsiung J Med Sci.

[CR15] Longa EZ, Weinstein PR, Carlson S, Cummins R (1989). Reversible middle cerebral artery occlusion without craniectomy in rats. Stroke.

[CR16] Luan H, Kan Z, Xu Y, Lv C, Jiang W (2013). Rosmarinic acid protects against experimental diabetes with cerebral ischemia: relation to inflammation response. J Neuroinflammation.

[CR17] Ma S, Wang J, Wang Y, Dai X, Xu F, Gao X, Johnson J, Xu N, Leak RK, Hu X, Luo Y, Chen J (2018). Diabetes mellitus impairs white matter repair and long-term functional deficits after cerebral ischemia. Stroke.

[CR18] Ni W, Zhang Y, Zhan Z, Ye F, Liang Y, Huang J, Chen K, Chen L, Ding Y (2017). A novel lncRNA uc.134 represses hepatocellular carcinoma progression by inhibiting CUL4A-mediated ubiquitination of LATS1. J Hematol Oncol..

[CR19] Ni W, Yao S, Zhou Y, Liu Y, Huang P, Zhou A, Liu J, Che L, Li J (2019). Long noncoding RNA GAS5 inhibits progression of colorectal cancer by interacting with and triggering YAP phosphorylation and degradation and is negatively regulated by the m6A reader YTHDF3. Mol Cancer.

[CR20] Park DJ, Koh PO (2018). Diabetes aggravates decreases in hippocalcin and parvalbumin expression in focal cerebral ischemia. Neurosci Lett.

[CR21] Pinto A, Di Raimondo D, Tuttolomondo A, Fernandez P, Arnao V, Licata G (2006). Twenty-four hour ambulatory blood pressure monitoring to evaluate effects on blood pressure of physical activity in hypertensive patients. Clin J Sport Med.

[CR22] Shupletsova JS, Bashmakova NV, Putilova NV, Deryabina EG, Tretyakova TB, Pestryaeva LA (2017). Risk factors of cerebral ischemia in infants born to mothers with gestational diabetes. Gynecol Endocrinol.

[CR23] Su EJ, Cao C, Fredriksson L, Nilsson I, Stefanitsch C, Stevenson TK (2017). Microglial-mediated PDGF-CC activation increases cerebrovascular permeability during ischemic stroke. Acta Neuropathol.

[CR24] Tu Y, Guo C, Song F, Huo Y, Geng Y, Guo M (2019). Mild hypothermia alleviates diabetes aggravated cerebral ischemic injury via activating autophagy and inhibiting pyroptosis. Brain Res Bull.

[CR25] Tuttolomondo A, Pedone C, Pinto A, Di Raimondo D, Fernandez P, Di Sciacca R, Licata G, Gruppo Italiano di Farmacoepidemiologia dell'Anziano (GIFA) researchers (2008). Predictors of outcome in acute ischemic cerebrovascular syndromes: The GIFA study. Int J Cardiol..

[CR26] Wang LQ, Zhou HJ (2018). LncRNA MALAT1 promotes high glucose-induced inflammatory response of microglial cells via provoking MyD88/IRAK1/TRAF6 signaling. Sci Rep.

[CR27] Wang S, Han X, Mao Z, Xin Y, Maharjan S, Zhang B (2019). MALAT1 lncRNA induces autophagy and protects brain microvascular endothelial cells against oxygen-glucose deprivation by binding to miR-200c-3p and upregulating SIRT1 expression. Neuroscience.

[CR28] Wang K, Sun Q, Zhong X, Zeng M, Zeng H, Shi X, Li Z, Wang Y, Zhao Q, Shao F, Ding J (2020). Structural mechanism for GSDMD targeting by autoprocessed caspases in pyroptosis. Cell.

[CR29] Wu A, Sun W, Mou F (2021). lncRNAMALAT1 promotes high glucoseinduced H9C2 cardiomyocyte pyroptosis by downregulating miR1413p expression. Mol Med Rep.

[CR30] Xie Y, Huang Y, Ling X, Qin H, Wang M, Luo B (2020). Chemerin/CMKLR1 axis promotes inflammation and pyroptosis by activating NLRP3 inflammasome in diabetic cardiomyopathy rat. Front Physiol.

[CR31] Xu Y, Fang H, Xu Q, Xu C, Yang L, Huang C (2020). LncRNA GAS5 inhibits NLRP3 inflammasome activation-mediated pyroptosis in diabetic cardiomyopathy by targeting miR-34b-3p/AHR. Cell Cycle.

[CR32] Yu H, Pardoll D, Jove R (2009). STATs in cancer inflammation and immunity: a leading role for STAT3. Nat Rev Cancer.

[CR33] Yu X, Ma X, Lin W, Xu Q, Zhou H, Kuang H (2021). Long noncoding RNA MIAT regulates primary human retinal pericyte pyroptosis by modulating miR-342-3p targeting of CASP1 in diabetic retinopathy. Exp Eye Res.

[CR34] Zhan JF, Huang HW, Huang C, Hu LL, Xu WW (2020). Long non-coding RNA NEAT1 regulates pyroptosis in diabetic nephropathy via mediating the miR-34c/NLRP3 axis. Kidney Blood Press Res.

[CR35] Zhang T, Wang H, Li Q, Fu J, Huang J, Zhao Y (2018). MALAT1 activates the P53 signaling pathway by regulating MDM2 to promote ischemic stroke. Cell Physiol Biochem.

[CR36] Zhang F, Zhao Q, Jiang Y, Liu N, Liu Q, Shi FD, Hao J, Xu Y, Lo EH, Wang X (2019). Augmented brain infiltration and activation of leukocytes after cerebral ischemia in type 2 diabetic mice. Front Immunol.

[CR37] Zhang G, Wang Q, Su D, Xie Y (2020). Long non-coding RNAMALAT1 knockdown alleviates cerebral ischemia/reperfusion injury of rats through regulating the miR-375/PDE4D axis. Front Neurol.

[CR38] Zhou HJ, Wang LQ, Xu QS, Fan ZX, Zhu Y, Jiang H (2016). Downregulation of miR-199b promotes the acute spinal cord injury through IKKbeta-NF-kappaB signaling pathway activating microglial cells. Exp Cell Res.

[CR39] Zhou X, Wang Q, Nie L, Zhang P, Zhao P, Yuan Q (2020). Metformin ameliorates the NLPP3 inflammasome mediated pyroptosis by inhibiting the expression of NEK7 in diabetic periodontitis. Arch Oral Biol.

