# Supplementary figures and images for: MALAT1 knockdown alleviates the pyroptosis of microglias in diabetic cerebral ischemia via regulating STAT1 mediated NLRP3 transcription
Source: Mol Med. 2023 Apr 3;29:44. doi: 10.1186/s10020-023-00637-2 (PMC10069069; doi:10.1186/s10020-023-00637-2)

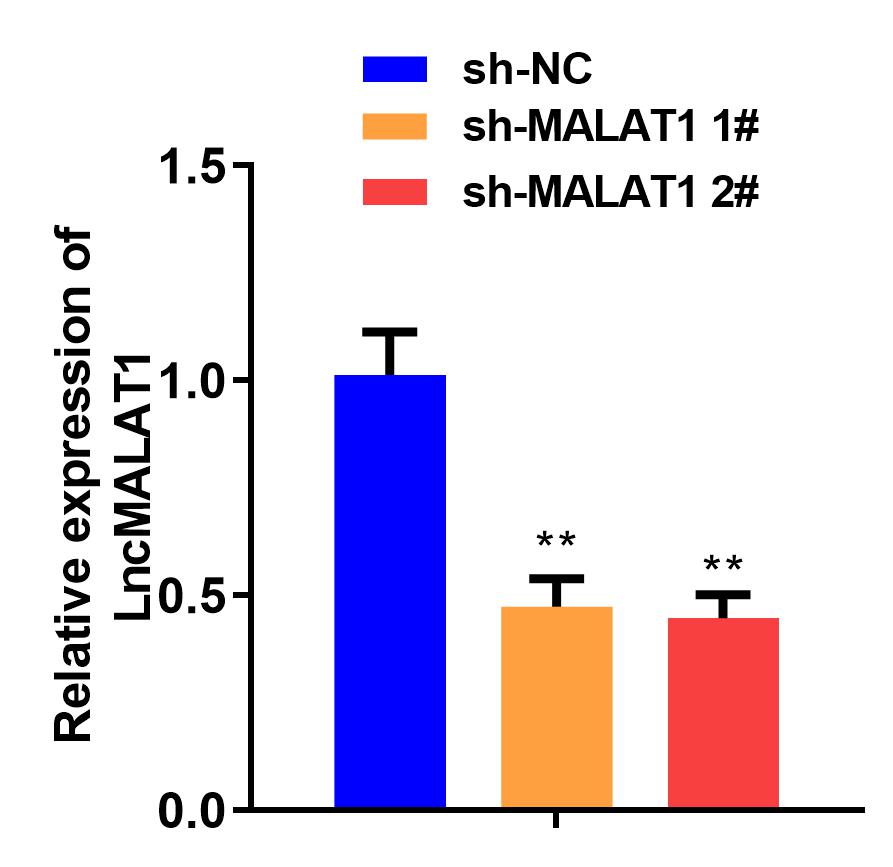

Supplement: Supplementary file 1 — Additional file 1: Figure 1. Verification of transfection efficiency of sh-MALAT1. The MALAT1 levels were detected by RT-qPCR after sh-MALAT 1# and sh-MALAT 2# transfection. **P<0.01. [file 10020_2023_637_MOESM1_ESM.jpg]

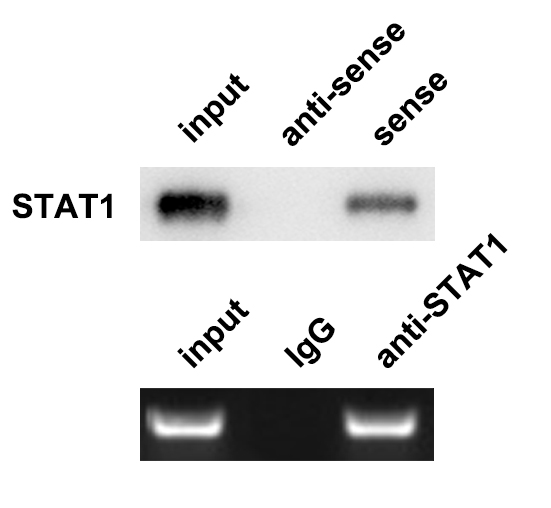

Supplement: Supplementary file 2 — Additional file 2: Figure 2. Verification of the interaction between MALAT1 and STAT1 in the HG treated cells. In the HG treated cells, the interaction between MALAT1 and STAT1 was also confirmed by RNA pull down assay. [file 10020_2023_637_MOESM2_ESM.jpg]

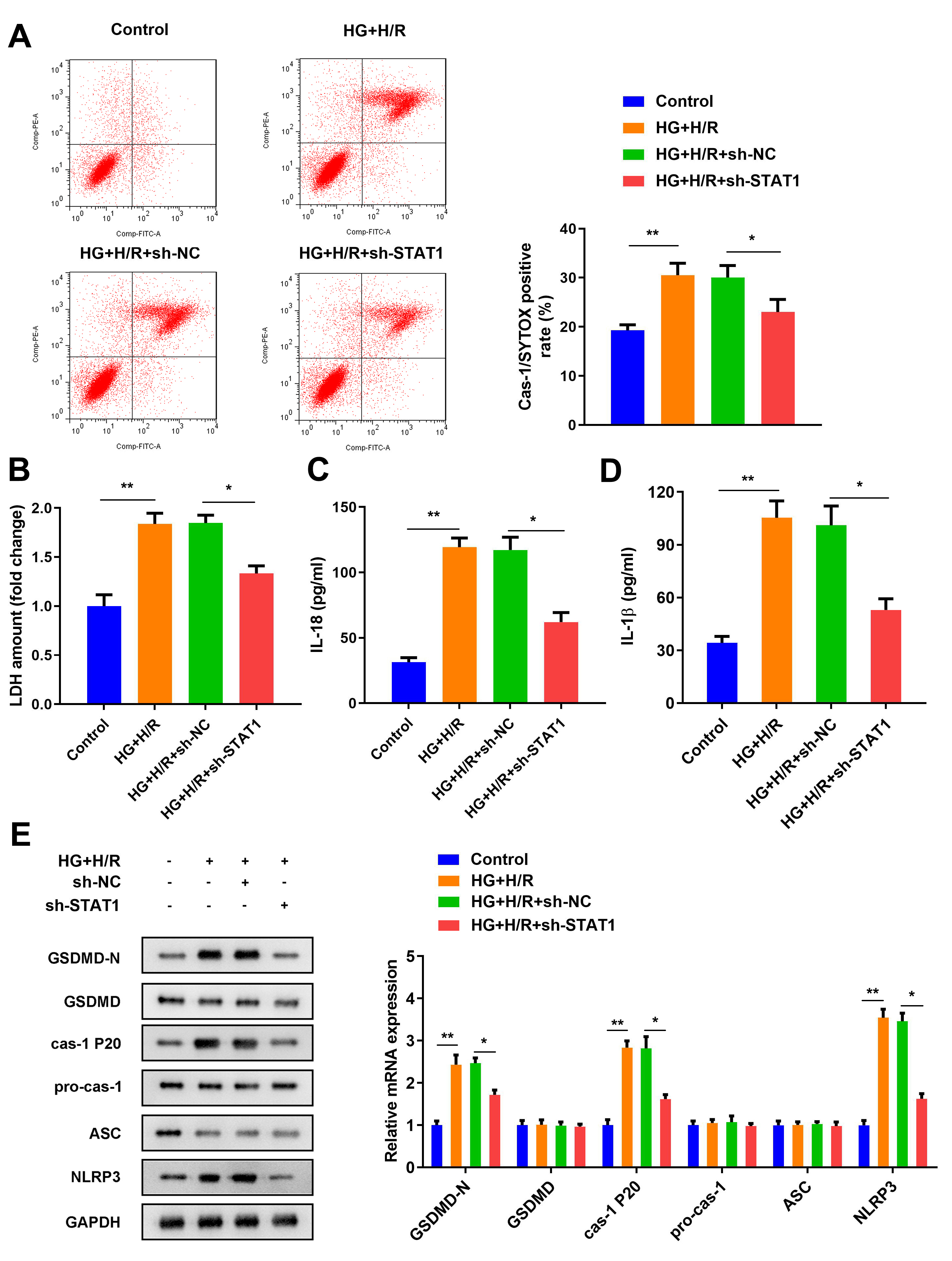

Supplement: Supplementary file 3 — Additional file 3: Figure 3. Knockdown of STAT1 decreased pyroptosis rate in high glucose and H/R treated BV2 cells. (A) after the indicated treatment, BV2 cells were labelled with caspase-1 antibody and SYTOX, and counted by flow cytometry.(B) LDH concentration of the BV2 cells were measured by ELISA kit after indicated treatment. The concentrations of IL-18 (C), and IL-1β (D) in the BV2 cells were measured by ELISA kit. (E) Western blot for GSDMD-N, GSDMD, caspase-1 p20, pro-caspase-1, ASC and NLRP3 of the BV2 cells 72 h after they received indicated treatment. (n = 6), *P<0.05, **P<0.01. [file 10020_2023_637_MOESM3_ESM.jpg]
